# Why the Philadelphia County Medical Society Is a Secret Society

**Published:** 1879-01

**Authors:** 


WHY THE PHILADELPHIA COUNTY MEDICAL SOCIETY IS A SECRET SOCIETY.—The Philadelphia County Medical Society, however efficient in many directions, is in one respect a standing discredit to the profession of medicine. It sits with closed doors, refuses admittance to reporters, and even forbids its own members from giving so much as an epitome of the scientific papers read before it to the general professional public. We venture to say that not only no other medical society, but no other professedly scientific association in the world, is equally narrow and short-sighted. And what is the "true inwardness" of this unheard-of action? We know it, and shall divulge it for the benefit of those medical journals at a distance who have expressed surprise at such a state of things (as well they might). There is a medical journal, not often heard of, in this city which, having failed in every other way to attain even a local circulation, its publishers thought might have some life galvanized into it as an "organ." Its editor, with a few active friends, one or two of them, we are sorry to add, officers of the society, rushed through a resolution one summer afternoon, that the "organ" should be the exclusive one, and the measure is carried out as has been noted. The poor-befooled members do not get a cent for their articles, the publisher having stipulated that he was to own them, but not to pay for them! Their papers attain a circulation of only a few hundred copies, around town principally; and as the journal in question is not of very frequent issue, and would be pretty sure to be behind time anyhow, of course, the embargo must be stringently enforced on more wide-awake periodicals. This is the explanation of the matter, and what a story it is to tell of the principal medical society of this city!—*Med. and Surg. Reporter.*

